# Proper mechanical stress promotes femoral head recovery from steroid-induced osteonecrosis in rats through the OPG/RANK/RANKL system

**DOI:** 10.1186/s12891-020-03301-6

**Published:** 2020-05-02

**Authors:** Dapeng Fu, Kairong Qin, Sheng Yang, Jianmin Lu, Haoyi Lian, Dewei Zhao

**Affiliations:** 1 Department of Biomedical engineering, Dalian University of Technology, Dalian, 116024 Liaoning People’s Republic of China; 2 Department of Orthopaedics, Affiliated Zhongshan Hospital of Dalian University, No. 6 Jiefang Street, Zhongshan District, Dalian, 116001 Liaoning People’s Republic of China

**Keywords:** Mechanical stress, Osteonecrosis of femoral head, OPG/RANK/RANKL, Bone density, Osteogenesis, Bone recovery

## Abstract

**Background:**

Long-term use of steroid may lead to osteonecrosis of the femoral head (ONFH). Mechanical stress may help bone formation and remodeling. This study aimed to probe the role of mechanical stress in the femoral head recovery in rats.

**Methods:**

Rat models with ONFH were induced by steroid. Rats were subjected to different levels of mechanical stress (weight-bearing training), and then the morphology and bone density of femoral head of rats were measured. The mRNA and protein levels of the OPG/RANK/RANKL axis in rat femoral head were assessed. Gain- and loss-of function experiments of OPG were performed to identify its role in femoral head recovery following stress implement. The ex vivo cells were extracted and the effects of stress and OPG on osteogenesis in vitro were explored.

**Results:**

Steroid-induced ONFH rats showed decreased bone density and increased bone spaces, as well as necrotic cell colonies and many cavities in the cortical bones and trabeculars. Proper mechanical stress or upregulation of OPG led to decreased RANK/RANKL expression and promoted femoral head recovery from steroid-induced osteonecrosis. However, excessive mechanical stress might impose too much load on the femurs thus leading even retard femoral head recovery process. In addition, the in vitro experimental results supported that proper stress and overexpression of OPG increased the osteogenesis of ex vivo cells of femoral head.

**Conclusion:**

This study provided evidence that proper mechanical stress promoted femoral head recovery from steroid-induced osteonecrosis through the OPG/RANK/RANKL system, while overload might inhibit the recovery process. This study may offer novel insights for ONFH treatment.

## Background

Steroid-induced osteonecrosis of the femoral head (ONFH) is a progressive disease caused by long-term use of hormone drugs, which results in impaired blood supply in the femoral head and causes bone marrow and osteocyte death, leading to internal structural disorders, and dysfunction of femoral head and hip joint [1, 2]. Aside from long-term use of chronic steroid, smoking, alcoholism, hip traumas, and prior hip surgery are also risk factors for ONFH [3]. The occurrence of ONFH has been rising in the past few years because of the wide application of steroid drugs for immunosuppression, particularly in the intervention of rheumatic diseases and in transplant patients [4]. Relative moderate core decompression may be effective in early-stage ONFH, and more major procedures including femoral osteotomy, bone grafting and even total hip replacement are needed for ONFH clinical intervention [5]. The development of this disorder may seriously affect the life quality of the patients [6]. Thus, understanding the pathogenesis and developing therapeutic approaches to ONFH is of great importance.

Bone is capable to react to mechanical changes by adjusting its internal microstructure via bone forming and resorbing cells, which is called bone modeling and remodeling [7]. Bone homeostasis is mediated by the remodeling process controlled by three cell types: osteoclasts, osteoblasts and osteocytes [8]. Mechanical stresses include mechanical strain, compressive stress and shear stress [9]. Mechanical stress stimulation acts as a key regulator in bone remodeling and formation and the stimulation [10]. Load loss may lead to deterioration of mechanical properties of bones and reduction in muscle strength and postural stability [11]. Mechanical stress has been suggested to promote osteoblast proliferation and attachment [12]. Therefore, mechanical stress may also have protective effects on ONFH.

Researches on pathological and physiological mechanisms of osteoprotegerin (OPG), receptor activator of nuclear factor kappa B (RANK) and RANK ligand (RANKL) provide new options for the ONFH therapy [13]. OPG, RANK and RANKL constitute a molecular triad that is closely correlated with vascular calcification, bone metabolism and immune system development through dendritic cells [14]. OPG and RANKL are two main regulators controlling activities of mature osteoclasts and osteoclastogenesis [15]. Based on these findings, we hypothesized that mechanical stress might exert protective functions in ONFH, where the OPG/RANK/RANKL trial system may play key roles. To validate this hypothesis, in vivo experiments on rats and in vitro experiments on ex vivo cells of femoral head were performed.

## Methods

### Ethics statement

The study was ratified by the Clinical Ethical Committee of Affiliated Zhongshan Hospital of Dalian University. All procedures were performed according to the ethical guidelines for the study of experimental pain in conscious animals. Great efforts were made to minimize the suffering of conscious animals.

### Establishment of steroid-induced osteonecrosis of femoral head (ONFH) rat models

A total of 140 sprague dawley rats (SYXK (Liaoning) 2017–0005) were included in this study, among which 10 rats were allocated into normal group, and the rest 130 rats were used for ONFH model establishment. After 1 week of adaptive feeding, the rats were weighed and intraperitoneally injected with 20 mg/kg lipopolysaccharide (LPS, Sigma-Aldrich, Merck KGaA, Darmstadt, Germany) for twice at a 24-h interval. Next, 24 h after the last LPS injection, the rats were further intramuscularly injected with methylprednisolone sodium succinate (40 mg/kg, Pfizer Company, Dalian, Liaoning, China) every 24 h for a total of 3 times. The normal rats were administrated with saline through intramuscular injection as controls. Six weeks after the last injection, 10 rats from the control group and the model group were collected and each rat was intraperitoneally injected with 800 mg/kg excess pentobarbital sodium for euthanasia [16]. After the injection, loss of righting reflex of rats was evaluated to verify that rats were unconscious, and cessation of heartbeat was verified by auscultation of left thoracic cavity of rats using a stethoscope. After confirmation of euthanasia, the bilateral femurs in 5 rats were collected for histomorphology measurement and the rest 5 rats were screened by Micro Computed Tomography (CT) to identify the model establishment. Among the included rats, 100 ones were successfully modeled and the ONFH rate was 76.9%.

### Construction of the lentiviral vectors

The lentiviral vectors pLVX-IRES-ZsGreen1 [containing green fluorescent protein (GFP) reporter gene] were purchased from Clonetch Inc. (CA, USA). The cDNA of OPG was synthesized by Sangon Biotech Co., Ltd. (Shanghai, China), and a marker sequence was inserted to the C-terminus of the cDNA to construct recombined OPG DNA. Then the DNA was cloned to the pLVX-OPG-ZsGreen1 to construct pLVX-OPG-IRES-ZsGreen1 vector, namely OPG vector, and the corresponding pLVX-IRES-ZsGreen1 empty vector was constructed and named as Mock.

The LV-OPG-RNAi-1 vector which contained small interfering RNA (siRNA) targeting OPG expression was constructed by Shanghai Genechem Co., Ltd. (Shanghai, China) and named as si-OPG, and the non-targeting lentiviral vector U6-MCS-Ubiquitin-EGFP-IRES-puromvcin was constructed as a control.

### Animal grouping and treatments

Next, 60 model rats were collected and randomly allocated into Sham group (*n* = 20, rats without weight-bearing), Partial group (*n* = 20, rats with partial weight bearing) and Total group (*n* = 20, rats with total weight bearing). Following 4 weeks of weight-bearing training, 10 rats in each group were euthanized as abovementioned methods for the following experiments, while the rest 10 rats were trained for 4 more weeks and then euthanized for experiments.

As to the remaining 30 model rats, 10 of them were allocated into the lentiviral vector (Lv)-mock group (rats injected with 100 ng Lv-mock vector through the bilateral ankle joints), 10 in the Lv-OPG group (rats injected with 100 ng Lv-OPG vector through the bilateral ankle joints), and the last 10 in the Lv-si-OPG group (rats injected with 100 ng Lv-si-OPG vector through the bilateral ankle joints). Following 8 weeks of partial weight-bearing training, rats in each group were further injected with corresponding vectors through the bilateral ankle joints for twice at a 2-week interval, and the rats were euthanized 4 weeks after the last injection.

All model rats were subjected to 1-weel adaptive training with 30 min each day. Rats were set on a treadmill with the speed set at 10 m/min and the inclination at 0°. Weight-bearing was introduced by strapping a strip load on the rat back. The load for rats in the Total group was 50% of the maximum bearing weight, and that for rats in the Partial group was 30% of the maximum bearing weight. Then the 8-week weight-bearing training was performed with the speed set at 15 m/min and the inclination at 0°. Rats underwent 6 cycles of 2-min running and 2-min relaxing each day for a total of 8 weeks. At the 4th and 8th week, 5 rats in each group were screened by Micro computed tomography (CT) and had the bone density evaluated, and the left-side femoral head of the rest 5 rats was used for reverse transcription quantitative polymerase chain reaction (RT-qPCR) and western blot analysis, while the right-side femoral head was used for tissue section preparation.

### Micro CT

Micro CT scanning was performed by the Institute of Laboratory Animal Sciences (Beijing, China) using an Inveon micro positron emission tomographic (PET)/CT instrument (Siemens Ltd., Erlangen, Germany) on the 5-cm near end of the femur. Then the bone volume/total volume (BV/TV), bone surface area/bone volume (BS/BV), trabecular thickness (Tb. Th) and the trabecular number (Tb. N) were evaluated.

### Hematoxylin and eosin (HE) staining

The tissues of femoral head were stained with HE as previously described [17]. Each experiment was performed in triplicate, and each section was observed under a microscope with 5 random fields selected.

### Immunohistochemistry

Femoral head tissue sections were collected and incubated with the antibodies against the following antigens vascular endothelial growth factor (VEGF, 1:500, ab15580), B-cell lymphoma-2 (Bcl-2, ab59348, 1:100) and caspase-3 (ab13847, 1:100) (all purchased from Abcam Inc., Cambridge, MA, USA) for 30 min. Then the sections were washed with phosphate buffer saline (PBS) for 3 times and incubated with 40 μL horseradish peroxidase-labeled streptavidin-working solution at 37 °C for 15 min. Following 3 times of PBS washes, the sections were stained with diaminobenzidine for color development, washed with distilled water, counterstained with hematoxylin for 30 s, dehydrated, sealed with neutral balsam, and finally observed under the microscope with 5 non-overlapping fields selected.

### RT-qPCR

Total RNA from tissues and cells was extracted using RNAiso Plus (Takara, Otsa, Shiga, Japan) and Trizol LS Reagent (Takara), respectively. Then the high quality of the extracted RNA was confirmed using formaldehyde denaturing electrophoresis. The reverse transcription PCR was then performed according to the instructions of a PrimeScript™ kit (Takara), and real-time qPCR was conducted on a SYBR-Premix Ex Taq (Takara). The mRNA expression was quantified with glyceraldehyde-3-phosphate dehydrogenase as the internal reference. The primers are shown in Table 1.

**Table 1:** Primer sequences for RT-qPCR

Primer	Sequence (5′-3′)
OPG	F: TTTGCCTGGGACCAAAGTGAATGCAGAGAG
	R: AGAAATGATAGGGAATCAGGTTCAATCAGT
RANK	F: TGGACAACCCAGGAAACCTTTCCTCCAAAA
	R: GCCAGCCGAGACTACGGCAAGTACCTGCGC
RANKL	F: GGCCA GGTGG TCTGC AGCATCGCTCTGTTC
	R: TTTATAGAATCCTGAGACTCCATGAAAACG
GAPDH	F: CGGACCAATACGACCAA
	R: AGCCACATCGCTCAGACACC

*RT-qPCR* Reverse transcription quantitative polymerase chain reaction, *OPG* Osteoprotegerin, *RANK* Receptor activator of nuclear factor kappa B, *RANKL* RANK ligand, *GAPDH* Glyceraldehyde-3-phosphate dehydrogenase, *F* Forward, *R* Reverse

### Western blot analysis

Total proteins from cells and tissues were extracted, and the concentrations were determined using a bicinchoninic acid kit (Qiagen GmbH, Hilden, Germany) according to the manufacturer’s protocol. The extracted proteins were run on sodium dodecyl sulfate polyacrylamide gel electrophoresis with the voltage increasing from 80 V to 120 V. Then the proteins were transferred onto polyvinylidene fluoride membranes using the semi-dry method at 80 mV for 30–45 min. The membranes were incubated with 5% bovine serum albumin at room temperature for 1 h, and then incubated with the primary antibodies against OPG (1:1000, ab2302), receptor activator of nuclear factor kappa B (RANK, 1:1000, ab32370), RANK ligand (RANKL, 1:5000, ab32064) and β-actin (1:5000, ab227387) (all purchased from Abcam) at 4 °C overnight. Afterwards, the membranes were washed with tris-buffered saline tween (TBST) (3 × 5 min), and incubated with the corresponding secondary antibody horseradish peroxidase-labeled immunoglobulin G (ab6747, 1:10000, Abcam) at room temperature for 1 h, After 3 times of TBST washes (5 min for each), the bands were visualized using chemiluminescence reagent on a Bio-Rad Gel Dol EZ imager (Bio-Rad Laboratories, California, USA). The target band was analyzed by calculating the gray value using ImageJ software (National Institutes of Health, Bethesda, Maryland, USA).

### Terminal deoxynucleotidyl transferase (TdT)-mediated dUTP nick end labeling (TUNEL)

Cell apoptosis in femoral head tissues was measured using a TUNEL kit (Roche Ltd., Basel, Switzerland) in accordance with the instructions [18]. The TUNEL-positive cells (apoptotic cells) showed stained condensed nuclei under the microscope.

### Separation and culture of ex vivo cells of femoral head

When rats were euthanized, the femoral head was collected under sterile conditions with the attached periosteum and the surrounding connective tissues removed. Then the femoral head was washed twice with PBS, and low-glucose Dulbecco’s modified Eagle medium (DMEM) was injected into the bone marrow to produce bone marrow stromal cell suspension. The suspension was then sorted in culture bottles and incubated in an incubator with 5% CO_2_ and saturated humidity. On the 2nd d, half of the DMEM was refreshed, and the medium was further refreshed once every 3 d after that. When the cell confluence reached 80% on the 10th d, the cells were detached with 0.25% trypsin and subcultured.

### Cell grouping and treatments

After extraction of ex vivo cells of femoral head, the cells were cultured in low-glucose DMEM containing 10% fetal bovine serum and 100 U/mL penicillin and 100 mg/mL streptomycin. The medium was further added with 8–10 mol-L dexamethasone, 10 mmol/L sodium β-glycerophosphate and 50 μg/mL vitamin C for cell subculture. The cells at passage 3 were harvested for subsequent experiments.

The cells were allocated into non-stressed group (0 g), 100 g-stressed group (corresponding to 978 rpm), 200 g-stressed group (1372 rpm), 400 g-stressed group (1941 rpm), Lv-mock group (cells were transfected with 100 ng Lv-mock vector and given 200 g stress), Lv-OPG group (cells were transfected with 100 ng Lv-OPG vector and given 200 g stress) and Lv-si-OPG group (cells were transfected with 100 ng Lv-si-OPG vector and given 200 g stress). The in vitro mechanical stimulation experiment was designed based on previous reports [19, 20]. The cells were respectively centrifuged for 30 min, 60 min and 120 min and further cultured. The cells were collected 48 h later for the following experiments.

### Measurement of alkaline phosphatase (ALP) activity

The activity of ALP was detected by ALP staining. All procedures were conducted as previously described [21].

### Alizarin red staining

Alizarin red staining was performed to measure the number of ex vivo cells of femoral head -differentiated calcified nodules, with the procedures guided by a former study [22].

### Immunofluorescence staining

Cells on the slides were rinsed 3 times with PBS, fixed in 4% paraformaldehyde at 4 °C for 15 min, and treated with 0.5% Triton-100 X for 20 min. Then the cell slides were incubated with the primary antibodies against osteocalcin (1:200, ab92552, Abcam) and runt-related gene 2 (RUNX2, 1:100, ab133504, Abcam) at 4 °C overnight. Next, the cells were washed with PBS and incubated with Alexa Fluora or fluorescein isothiocyanate-labeled goat-anti rabbit secondary antibody (1:5000, ab150088, Abcam) at 37 for 1 h. The nuclei were stained with 4′,6-diamidino-2-phenylindole, and the cells were observed under a fluorescence microscope (DM3000, Leica Biosystems, Shanghai, China).

### Statistical analysis

SPSS 21.0 software (IBM Corp. Armonk, NY, USA) was applied for data analysis. The Kolmogorov-Smirnov test was used to determine whether data were in normal distribution. Measurement data were described as mean ± standard deviation (SD). Differences between two groups were measured using the *t*-test, whereas the differences among multiple groups were analyzed using one-way or two-way analysis of variance (ANOVA), followed by pairwise comparisons using Tukey’s multiple comparisons test. The *p* value was calculated using a two-tailed test, and *p* < 0.05 indicated a significant difference.

## Results

### Successful establishment of steroid-induced ONFH rat models

**Fig. 1:**
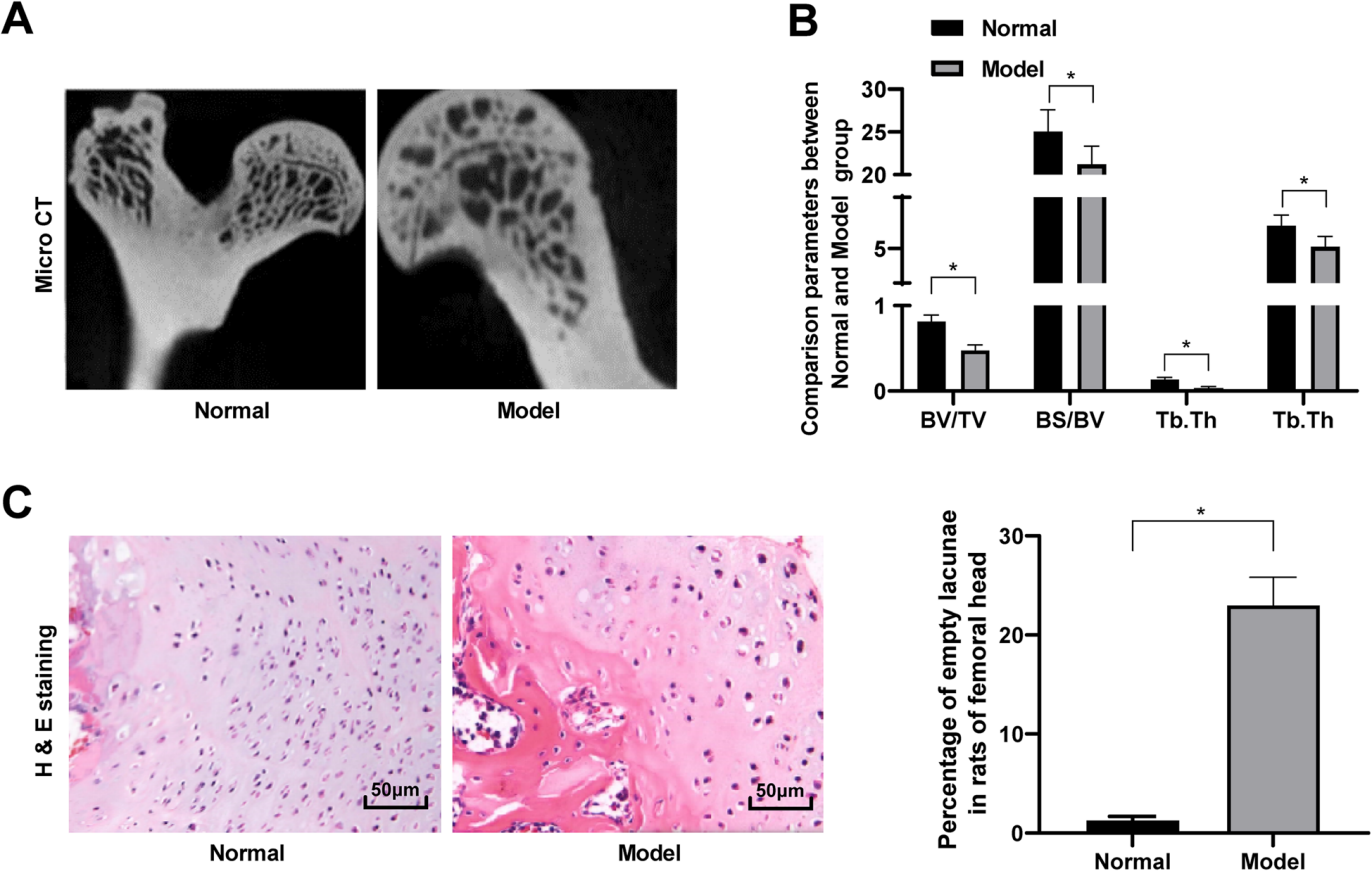
Establishment of steroid-induced ONFH rat models. **a**-**b**, Micro CT diagnosis was performed to determine the parameters of trabecular in the cancellous bone of the femoral head between the normal and model groups; **c**, HE staining was performed to determine the morphology changes in the femoral head. Data are expressed as mean ± SD; in panels **a** and **b**, two-way ANOVA and Tukey’s multiple comparisons test was used for data analysis, while in panel **c**, data were analyzed using the unpaired *t* test; *p < 0.05; Replicates = 3, *n* = 5 in each group

During the feeding period, normal rats showed healthy physical conditions, frequent food intake, good mental condition and quick responses. When captured, the rats had no abnormal urine or excrement. LPS and methylprednisolone sodium succinate were used to construct ONFH model in rats (the ONFH rate was 76.9%). Regarding the model group, the rats presented thin subcutaneous fat, decreased food and water intake, darkened hair with hair loss, bad mental condition with the susceptibility to stimulation, and impotent hind legs, and some rats even moderately limped. According to the femoral head morphology screened by Micro CT, the articular surface of the femoral head of normal rats was smooth, and the cartilages were clean, strong, solid and flexible, while the cartilages of the femoral head of model rats were in dark color without gloss (Fig. 1a), and the bones were fragile and easily detached from the surrounding tissues. In addition, BV/TV and BS/BV showed notable differences between the normal rats and the model rats, while Tb showed no significant difference (Fig. 1b). Thereafter, the femoral head tissues of rats were extracted and stained with HE. The cartilages of normal rats presented smooth surface, clear shape, even thickness, normal number, and normal connection between the calcified zone and trabecular. The cortical bones and trabecular also had clear osteocytes with occasional visible cavities. But in model rats, the cartilages were thin, and the cartilages surface became rough and uneven with partial surface lost. Besides, a large number of osteocytes and bone matrix decreased or lost, with necrotic cell colonies and many cavities appeared in the cortical bones and trabeculars (Fig. 1c).

### Proper mechanical stress promotes bone recovery from steroid-induced ONFH in rats

**Fig. 2:**
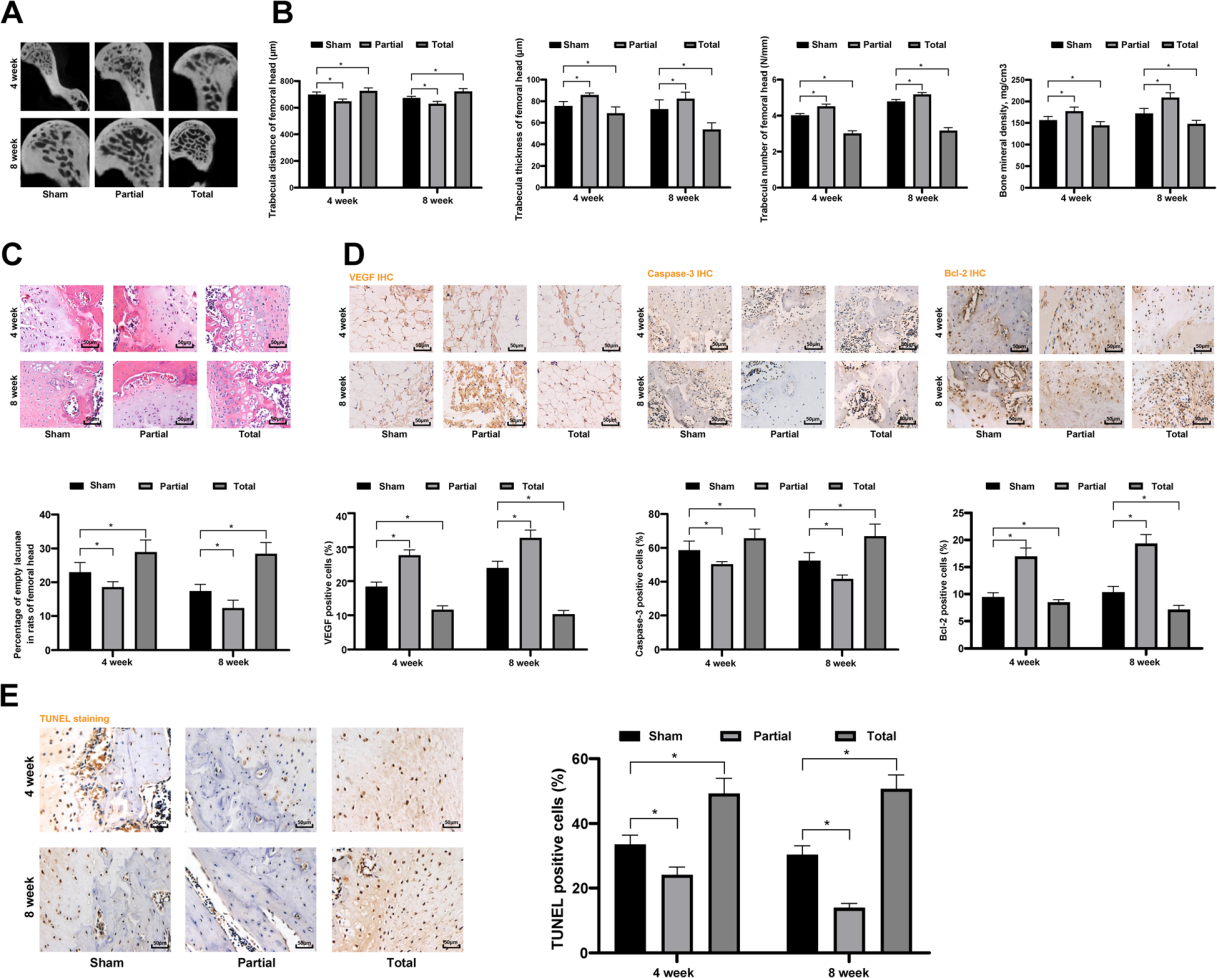
Proper mechanical stress promotes bone recovery from steroid-induced ONFH in rats. **a**-**b**, Micro CT diagnosis was performed to determine the parameters of trabecular in the cancellous bone of the femoral head between the normal and model groups; **c**, HE staining was performed to determine the morphology changes in the femoral head; **d**, immunohistochemistry was performed to determine VEGF, Bcl-2 and caspase-3 expression in the femoral head; **e**, TUNEL was performed to determine the apoptosis rate of cells in femoral head tissues. Data are expressed as mean ± SD; two-way ANOVA and Tukey’s multiple comparisons test was used to determine statistical significance; *p < 0.05; Replicates = 3, *n* = 5 in each group

The model rats were imposed with different mechanical stress (weight-bearing training) and correspondingly allocated into the Sham, Partial and Total groups as aforementioned. Micro CT was performed to measure the morphology of femoral head and bone density of rats. The results told that following 4 or 8 weeks of weight-bearing training, the trabecular number and the bone density were increased, and the trabecular spaces were decreased in rats both in the Sham and Partial groups, and the Partial group presented better trends than the Sham group. However, the rats in the Total group presented decreased trabecular number and bone density, but increased trabecular spaces in the femoral head (all *p* < 0.05) (Fig. 2a-b). The femoral head tissues were further stained with HE, which suggested that compared to the Total group, the bone lacuna rate was decreased in the Sham and Partial groups, especially in the Partial group (*p* < 0.001) (Fig. 2c). Next, immunohistochemistry of VEGF, Bcl-2 and caspase-3 was performed. The results showed that following 4 or 8 weeks of weight-bearing training, the positive rates of VEGF and Bcl-2 were increased while the caspase-3 positive rate was reduced in the Sham and Partial groups compared to the Total group, and the Partial group showed greater changes (Fig. 2d). Besides, TUNEL results told that the apoptotic cells in tissues in the Sham and Partial groups were notably reduced as compared to the Total group, in particular in the Partial group (Fig. 2e). Based on these results, proper stress could promote bone recovery from steroid-induced ONFH, while excessive stress might even inhibit the femoral head recovery.

### OPG is highly-expressed while RANKL is lowly-expressed under proper mechanical stress

**Fig. 3:**
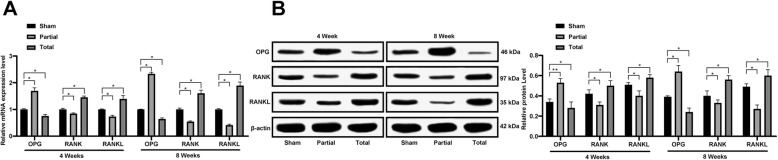
OPG is highly-expressed while RANKL is lowly-expressed under proper mechanical stress. **a**-**b**, relative mRNA (**a**) and protein (**b**) levels of OPG/RANK/RANKL in the femoral head tissues were measured using RT-qPCR and western blot analysis. Data are expressed as mean ± SD; two-way ANOVA and Tukey’s multiple comparisons test was used to determine statistical significance; *p < 0.05; Replicates = 3, *n* = 5 in each group

RT-qPCR and western blot analysis were performed to determine the mRNA and protein levels of OPG/RANK/RANKL in the femoral head tissues of each group, respectively. Following 4 or 8 weeks of weight-bearing training, the mRNA and protein levels of OPG were increased, while the levels of RANK/RANKL were decreased in the Sham and Partial groups compared to those in the Total group, and the changes were more intensive in the Partial group (all *p* < 0.05) (Fig. 3a-b).

### OPG silencing reverses the proper stress-promoted bone recovery from ONFH

**Fig. 4:**
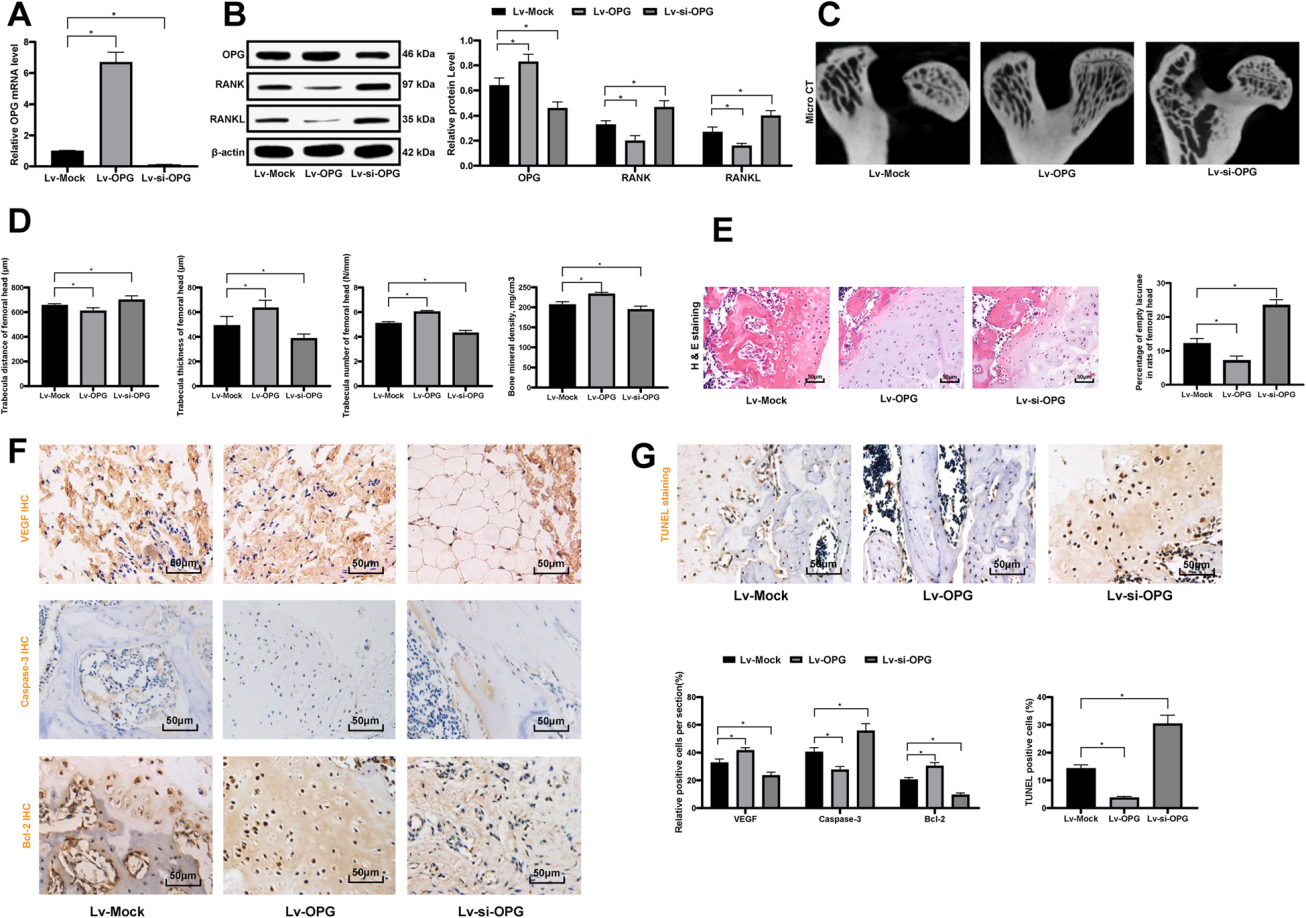
OPG silencing reverses the proper stress-promoted bone recovery from ONFH. **a**-**b**, mRNA (**a**) and protein (**b**) levels of OPG/RANK/RANKL were determined by RT-qPCR and western blot analysis, respectively; **c**-**d**, Micro CT diagnosis was performed to determine parameters of trabecular in the cancellous bone of the femoral head; **e**, HE staining was performed to determine the morphology changes in the femoral head; **f**, immunohistochemistry staining was performed to determine the expression of VEGF, Bcl-2 and Caspase-3 in femoral head tissues; **g**, TUNEL was performed to determine the apoptotic cells in femoral head tissues. Data are expressed as mean ± SD; one-way or two-way ANOVA and Tukey’s multiple comparisons test were used for data analysis; *p < 0.05; Replicates = 3, *n* = 5 in each group

To further confirm the roles of OPG in bone recovery following ONFH in rats, OPG overexpressing vector Lv-OPG and OPG silencing vector Lv-si-OPG were constructed and injected into the rats in the Partial group 8 weeks following weight-bearing training for twice at a 2-week interval. The rats were euthanized 4 weeks after the second injection, and the transfection efficiency was measured using RT-qPCR and western blot analysis by detection OPG levels (Fig. 4a-b). Then the trabecular number, trabecular spaces and the bone density of rats were screened using Micro CT. The results suggested that overexpression of OPG increased trabecular number and bone density and decreased the trabecular spaces, while silencing OPG, accordingly, led to reversed trends (Fig. 4c-d). Besides, the HE staining results revealed that overexpression of OPG decreased the bone lacuna rate in rat femoral head while OPG inhibition increased the rate (Fig. 4e). Moreover, the immunohistochemistry results identified that overexpression of OPG promoted the VEGF and Bcl-2 expression while inhibited caspase-3 expression (Fig. 4f), and TUNEL results suggested that overexpression of OPG inhibited the apoptosis of osteoblasts in rat femoral head (Fig. 4g), while downregulation of OPG reversed these changes (all *p* < 0.05).

### Proper mechanical stress promotes differentiation of ex vivo cells of femoral head

**Fig. 5:**
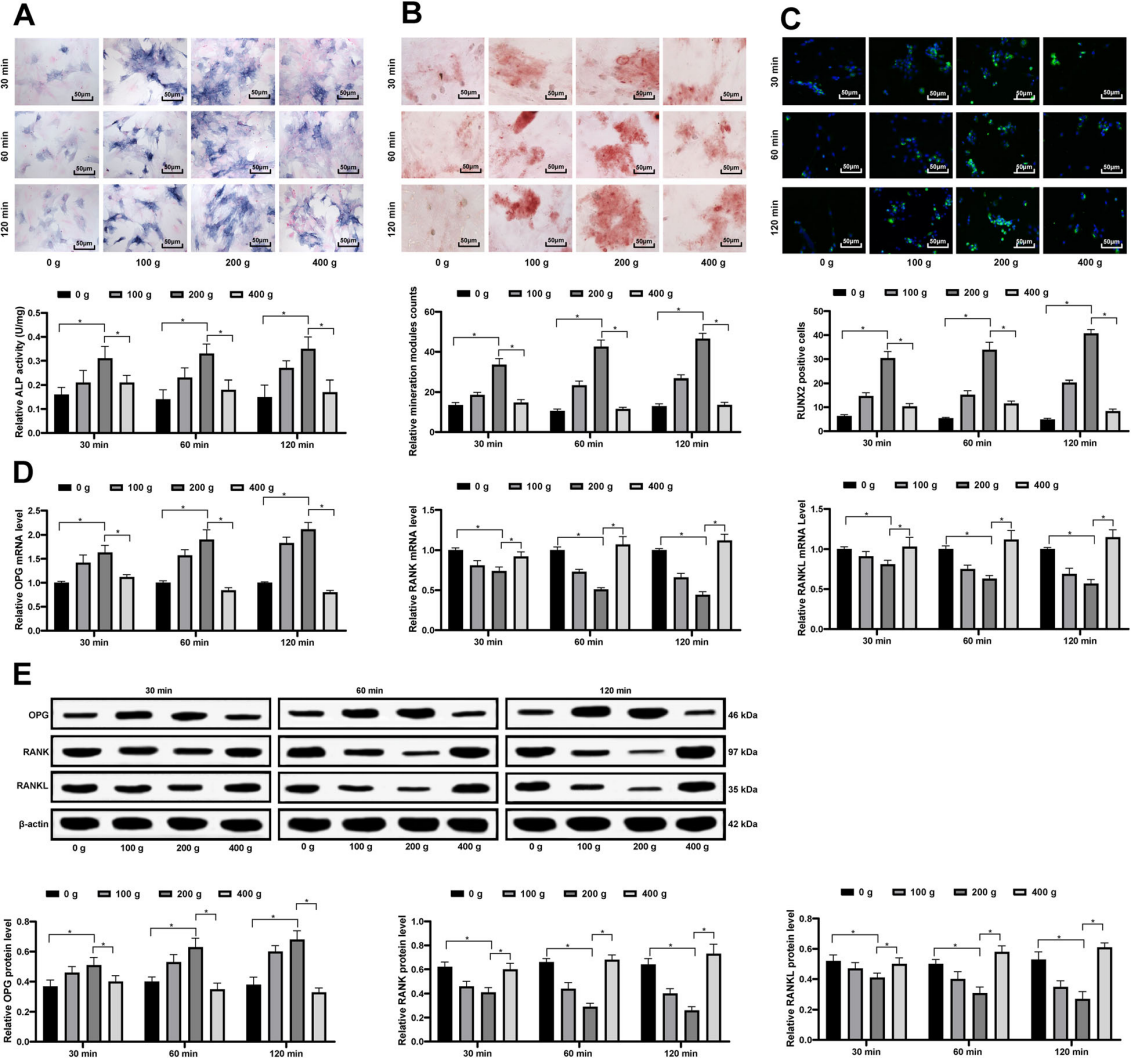
Proper mechanical stress promotes differentiation of osteoblasts. **a**-**c**, osteogenesis ability of ex vivo cells of femoral head was determined by ALP staining (**a**), alizarin red staining (**b**) and RUNX2 immunofluorescence staining (**c**); **d**-**e**, mRNA (**d**) and protein (**e**) levels of OPG/RANK/RANKL were determined by RT-qPCR and western blot analysis, respectively. Data are expressed as mean ± SD; two-way ANOVA and Tukey’s multiple comparisons test were used for data analysis; *p < 0.05; Replicates = 3

In vitro experiments were also performed by extracting the ex vivo cells of femoral head from model rats. The extracted cells were imposed with different mechanical stress as 0 g, 100 g (978 rpm), 200 g (1372 rpm) and 400 g (1941 rpm). Following 30, 60 and 120 min of centrifugation, the cells were further cultured for osteogenic differentiation. Next, the ALP activity and proliferation ability of cells were measured, and it was found that under a same stress time, the stressed cells showed increased ALP activity and proliferation ability, while the 200-g stress led to further promoted ALP activity and proliferation compared to the 400-g stress (Fig. 5a). The formation of calcified nodules was consistent with the ALP activity in osteoblasts according to the alizarin red staining (Fig. 5b). Next, immunofluorescence staining was conducted to measure the expression of osteogenic protein RUNX2 in cells, which suggested that proper stress elevated the protein level of RUNX2, while this elevation was reduced when there was excessive stress (like 400 g) (Fig. 5c). Moreover, RT-qPCR and western blot analysis were performed to identify the mRNA and protein levels of OPG/RANK/RANKL in cells, and the results suggested that proper stress (200 g) promoted the level of OPG and decreased the levels of RANK and RANKL, while the 400-g stress reversed these trends (Fig. 5d-e) (all *p* < 0.05).

### Silencing OPG reduces the mechanical stress-promoted osteogenesis of ex vivo cells of femoral head

**Fig. 6:**
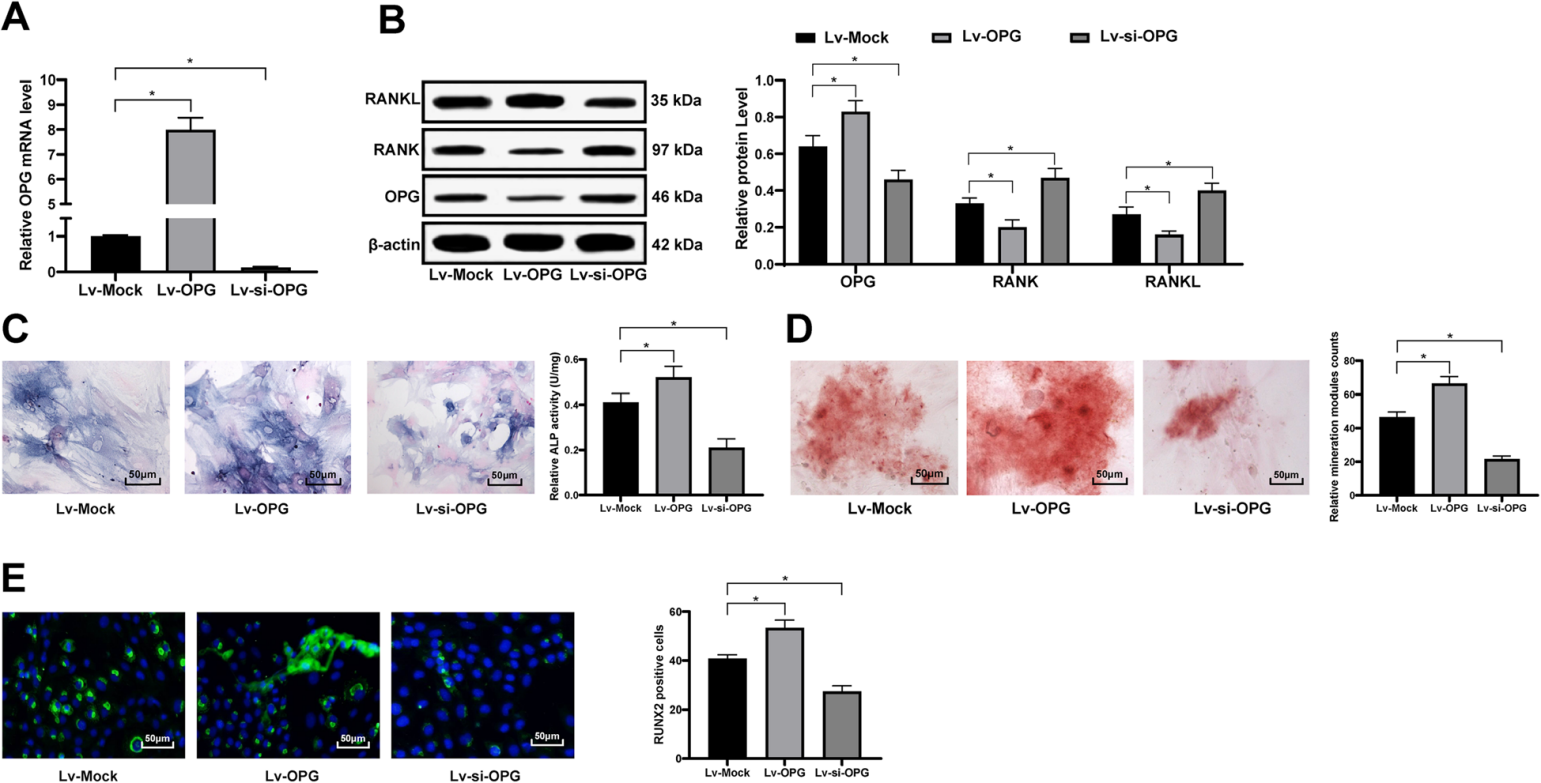
Silencing OPG reduces the mechanical stress-promoted osteogenesis. **a**-**b**, mRNA (**a**) and protein (**b**) levels of OPG/RANK/RANKL were determined by RT-qPCR and western blot analysis, respectively. **c**-**e**, osteogenesis ability of extracted ex vivo cells of femoral head was determined by ALP staining (**c**), alizarin red staining (**d**) and RUNX2 immunofluorescence staining (**e**). Data are expressed as mean ± SD; one-way or two-way ANOVA and Tukey’s multiple comparisons test were used for data analysis; *p < 0.05; Replicates = 3

The Lv-OPG and Lv-si-OPG vectors were transfected into the extracted and purified ex vivo cells of femoral head as well, with the successful transfections identified by RT-qPCR and western blot analysis (Fig. 6a-b). Then the cells were subjected to 200-g stress for 120 min and cultured for osteogenic differentiation. The results suggested that overexpression of OPG further promoted the osteogenesis of ex vivo cells of femoral head and inhibited cell apoptosis, while OPG inhibition partially reversed this promotion (Fig. 6c-e).

## Discussion

A variety of treating procedures have been implemented on ONFH; however, most of these procedures did not present satisfactory clinical outcomes, leaving great suffering to patients and great challenge in therapy development in this field [13]. Mechanical stress is considered to play a critical role in the pathological osteogenesis progression [23], and the OPG/RANK/RANKL trail system exerts key functions in bone metabolism, vascular calcification [14]. In the current study, we identified that proper mechanical stress could promote bone formation and femoral head recovery from osteonecrosis, while excessive stress could even impede the recovery, during which the OPG//RANK/RANKL system was involved.

Firstly, rat models with ONFH showed poor physical and mental conditions. The femur was fragile and easily separable, and the cartilages surface became rough and uneven with partial surface lost, and a large number of osteocytes and bone matrix were decreased or lost. Collapse of the articular surface and osteosyte death are typical outcomes of ONFH [3]. We found proper stress promoted femoral head recovery from osteonecrosis, and the bone density and trabecular number were increased as well. The trabecular number is an important parameter for bone strength assessment in everyday practice; in particular, the trabecular bone score has been developed as a reflection of bone mineral density and bone microarchitecture [24]. Mechanical stress has been suggested to regulate bone metabolism and promote bone growth [25]. More intuitively, physical activities and exercise are well-known to improve bone outcomes and strengthen hip and spine bone formation owing to the skeletal loading stimulation [26]. On the other hand, excessive stress reduced the recovery process with deteriorated symptoms. Concentration of immoderate mechanical stress may induce fatigue fractures or periprosthetic fractures [27]. Similarly, optimal mechanical stimulation was suggested to impede osteoarthritis progression, while increased stress may exacerbate osteoarthritis in contract [28]. Our study suggested that proper mechanical stress increased the levels of VEGF and Bcl-2 but decreased the caspase-3 expression in model rats. VEGF is a coordinate regulator drives angiogenesis, osteogenesis and skeletal growth [29, 30]. These findings suggested that proper mechanical stress promoted osteogenesis and bone recovery from the molecular perspective. Osteoblasts are important mechanical receptors for the mechanical stimuli to biochemical signal transformation, and they release bone matrix to induce bone matrix mineralization [9]. Aside from the above findings from in vivo experiments, we further extracted the ex vivo cells of femoral head and performed in vitro experiments. Centrifugal stresses were imposed on cells, after which the ALP activity and proliferation of ex vivo cells of femoral head, and the RUNX2 expression were increased. The ALP activity is an important marker of osteoblast differentiation [31]. RUNX2 mediates proliferation of osteoblast progenitors and regulates the differentiation into osteoblasts through multiple signaling molecules and transcription factors, and it is essential for osteoblast differentiation as well [32]. As in line with our findings, proper mechanical stress has been documented to increase the periostin production in osteoblasts, which may further inhibit osteoclast differentiation [8]. Herein, it can be inferred that proper stress could promote differentiation of ex vivo cells of femoral head and bone formation of rats with ONFH.

The OPG/RANK/RANKL axis acts as a key regulatory mechanism controlling the differentiation and activities of both osteoblasts and osteoclasts, thus maintaining bone homeostasis to prevent bone loss and ensure a normal bone turnover [33]. We further investigated the possible mechanisms involved in the above events. OPG and RANKL are main molecules considered to mediate the role of mechanical stress in bone formation [25]. Here, our study found that proper mechanical stress promoted OPG expression while decreased RANK/RANKL expression. Serum OPG levels were decreased in the rat models and THP-1 cells with steroid-induced necrosis of the femoral head, while RANK and RANKL levels were increased [34]. The OPG/RANK/RANKL system plays vital roles in bone remodeling, among which the RANK/RANKL interaction could promote proliferation and enhance the viability of osteoclast, while the stimulatory role of RANKL in osteoclast maturation and function could be suppressed by OPG, which serves as a decoy receptor for RANKL [13, 15, 34]. Likewise, excessive mechanical stress led to relatively decreased OPG while increased RANK/RANKL expression compared to proper stress, and the findings that inhibition on OPG reversed the promoting role of proper stress in femoral head recovery validated that the stress stimulates this recovery through the OPG/RANK/RANKL system. Soluble RANKL administration was documented to rapidly drive bone loss in mice through activation of bone resorption [35]. Whereas soluble OPG molecules bind to RANKL to block the binding of RANKL to RANK, thereby preventing osteoclast formation and maturation [36]. Interestingly, compressive force induced the upregulatino of RANKL/OPG ratio in human periodontal ligament cells [37]. Similarly, the in vitro experiments suggested that silencing OPG reversed the role of proper stress on the differentiation of ex vivo cells of femoral head. Compressive loading induced a 4.2-fold increase in RANKL gene expression, and OPG protein synthesis in the compressed cells was significantly decreased [38]. Osteonecrosis of the femoral head was observed both in the non-weight-bearing rats and in the weight-bearing rats, and non-traumatic ONFH developed in non-weight-bearing rats, indicating that weight bearing does not contribute to the development of non-traumatic ONFH in rats [39]. The effects of mechanical loading on the trabecular bone of the femoral head were not significant, suggesting that the effect of mechanical loading in the rats with backpack mainly occurs at cortical bone sites [40]. Studies show that chondrocytes are highly sensitive to mechanical stress stimulation, and the mechanical stimulation of normal physiological load can activate the synthesis and metabolism of chondrocytes and promote the formation and deposition of cartilage matrix [41, 42]. On the contrary, excessive mechanical load aggravates the catabolic process of articular chondrocytes, leading to the decrease of cell matrix formation, the increase of proteolytic enzyme activity and the apoptosis of chondrocytes [43].

## Conclusions

In conclusion, our study provided evidence that proper mechanical stress could improve osteoclast differentiation and promote femoral head recovery from steroid-induced osteonecrosis with the involvement of the OPG/RANK/RANKL trial system. These findings may provide novel insights into exercises and physical activities in ONFH recovery. We hope more studies will be carried out in the near future to identify our findings and to figure out more detailed molecular mechanisms in ONFH to develop more therapeutic options.

## Data Availability

All the data generated or analyzed during this study are included in this published article.

## Abbreviations

ALP: Alkaline phosphatase
ANOVA: Analysis of variance
Bcl-2: B-cell lymphoma-2
BS/BV: Bone surface area/bone volume
BV/TV: Bone volume/total volume
DAPI: 4′, 6-diamidino-2-phenylindole
DMEM: Dulbecco’s modified Eagle medium
GFP: Green fluorescent protein
HE: Hematoxylin and eosin
SD: Standard deviation
Micro CT: Micro Computed Tomography
ONFH: Osteonecrosis of femoral head
OPG: Osteoprotegerin
PBS: Phosphate buffer saline
RANK: Receptor activator of nuclear factor kappa B
RANKL: Receptor activator of nuclear factor kappa B ligand
RT-qPCR: Reverse transcription quantitative polymerase chain reaction
RUNX2: Runt-related gene 2
siRNA: small interfering RNA
Tb. N: Trabecular number
TBST: Tris-buffered saline tween
Tb. Th: Trabecular thickness
TUNEL: Terminal deoxynucleotidyl transferase (TdT)-mediated dUTP nick end labeling
